# Glucose release kinetics of different feed ingredients and their impact on short-term growth of pigs by influencing carbon-nitrogen supply synchronization

**DOI:** 10.1186/s40104-025-01198-6

**Published:** 2025-05-22

**Authors:** Mingyi Huang, Lei Xue, Yifan Wu, Qinzheng Sun, Yanwei Xu, Jia Li, Xiaoyi Yu, Yu Cao, Jingyi Huang, Zeyu Zhang, Jinbiao Zhao, Dandan Han, Defa Li, Junjun Wang

**Affiliations:** State Key Laboratory of Animal Nutrition and Feeding, College of Animal Science and Technology, China Agricultural University, Beijing, 100193 PR China

**Keywords:** Energy and nitrogen utilization, Glucose release kinetics, Growth performance, Pig, Synchronized nutrient supply

## Abstract

**Background:**

Pigs fed diets with different ingredients but identical nutritional levels show significant differences in growth performance, indicating that growth may also be influenced by the synchronicity of dietary carbon and nitrogen supply. Therefore, this study aimed to determine glucose release kinetics of various feed ingredients, to investigate a glucose release pattern that is conducive to synchronized carbon–nitrogen supply, and to elucidate the underlying mechanisms by which this synchronization optimizes growth of pigs.

**Results:**

We analyzed the glucose release kinetics of 23 feed ingredients in vitro and found that their glucose release rates and amounts varied greatly. Based on this, a nitrogen-free diet and 5 purified diets, which represented the observed variations in glucose release rates and quantities among feed ingredients, were designed for 18 ileal-cannulated pigs. The results demonstrated that slower glucose release pattern could disrupt the synchrony of dietary carbon and nitrogen supply, reducing the growth of pigs and increasing nitrogen losses. Specifically, the diet with slower and moderate amounts of glucose release showed a relatively slower release of amino acids. Pigs fed this diet had the lower amino acid digestibility and the enrichment of harmful bacteria, such as *Streptococcus*, in the terminal ileum. Conversely, the diets with slower and lower glucose release exhibited a relatively rapid release of amino acids but also resulted in poor growth. They increased glucogenic amino acid digestibility and potentially enriched bacteria involved in nitrogen cycling and carbon metabolism. Notably, only the diet with rapid glucose release achieved synchronized and rapid release of nutrients. Pigs fed this diet exhibited higher amino acid digestibility, decreased harmful bacteria enrichment, improved nutrient utilization, and enhanced short-term growth performance.

**Conclusions:**

Our research analyzed significant differences in glucose release kinetics among swine feed ingredients and revealed that slow glucose release disrupted dietary carbon–nitrogen supply synchrony, shifting amino acid utilization and enriching pathogens, negatively impacting growth and nutrient utilization. Consequently, choosing feed ingredients releasing glucose at a rapid rate to balance dietary carbon and nitrogen supply helps promote pig growth, and ensures efficient feed utilization.

**Supplementary Information:**

The online version contains supplementary material available at 10.1186/s40104-025-01198-6.

## Introduction

Diets with equivalent nutritional levels still resulted in significant differences in growth performance and protein utilization efficiency of pigs [1–3]. This demonstrates that changes in pig growth performance and protein utilization cannot be solely attributed to dietary nutritional levels. Instead, the timing and synchronicity of nutrient supply might be crucial factors underlying these variations [4, 5]. Unfortunately, these factors are often overlooked in common diet formulation practices. Thus, focusing on the release characteristics of nutrients may help us gain a deeper understanding of nutrient utilization and improve pig growth performance.

Glucose, as the primary nutrient providing energy for organisms, plays a crucial role in maintaining nutritional homeostasis. Recent studies have shown that different starches exhibit varying rates of glucose release [6, 7], which directly affects the balance and timing of dietary glucose supply. This variability can make it challenging to synchronize nutrient supply in diets. A comprehensive understanding of the glucose release characteristics of various feed ingredients and their impact on nutrient synchronization is essential for formulating optimal diets for pigs.

Furthermore, researchers have also found that adding crystalline amino acids can disrupt the balance between carbon and nitrogen supply [8, 9]. They discovered that using pea starch, which releases glucose slowly, could reduce the utilization of these amino acids in low-protein diets [3]. Conversely, cassava starch, which rapidly releases glucose, contributed to mitigating this effect [2]. These findings highlight the importance of managing glucose release to ensure the synchronicity of carbon and nitrogen supply [1, 10, 11]. However, there is still a gap in understanding how glucose release from starch digestion interacts with nitrogen-containing nutrients from protein digestion in combined feed ingredients [12–15]. Even without added crystalline amino acids, differences in the digestive characteristics of protein and starch can cause variations in the postprandial synchronization of dietary carbon and nitrogen [16]. As a result, the starch type deemed appropriate in some studies does not consistently promote the growth of growing-finishing pigs [3, 17–19]. Given this, the variations in synchronization affected by the combination of feed ingredients warrant further attention.

Currently, evidencing studies rely on qualitative descriptions or indirect measurements, which fail to provide a clear explanation of how glucose release affects the synchronous supply of carbon and nitrogen and its subsequent impact on growth performance. Therefore, there is a need for more research to fill these knowledge gaps and provide a clearer understanding of the interplay between glucose release and nutrient synchronization. Hence, we assessed the glucose release kinetics of 23 feed ingredients, providing a foundation for addressing nutrient supply synchronization in swine diets. Building on these insights, we prepared 5 purified diets, which represent different gradients in glucose release rate and amount. Key digestion intervals were targeted to capture and quantify variations in nutrient release synchronization in vitro. Ileal-cannulated pigs were employed to investigate the impact of the variation in carbon and nitrogen release synchronization on energy and nitrogen utilization efficiency and short-term growth performance.

## Materials and methods

### In vitro digestion of 23 feed ingredients and 5 diets

Before initiating the in vitro digestion, feed ingredients or diets were ground through a 1-mm sieve, and 0.5 g of each was weighed and placed into three separate centrifuge tubes as replicates. Then, the digestion procedure, which was slightly modified based on methods described in previous studies [6, 20, 21], was carried out in two successive digestive phases: gastric and intestinal phases. During the gastric phase, samples were diluted with 10 mL of simulated gastric juice and incubated for 2 h at 39 °C with shaking. The simulated gastric juice was 0.1 mol/L phosphate buffer at pH 3.5, containing pepsin (0.005 g/mL; P7000, Sigma-Aldrich, Saint Louis, USA) and guar gum (0.005 g/mL; G9310, Solarbio, Beijing, China). To prevent bacterial growth, 0.25 mL of chloramphenicol solution (0.5 g chloramphenicol per 100 mL ethanol; C8050, Solarbio, Beijing, China) was also added. At the end of this phase, 10 mL of 0.048 mol/L NaOH solution was added to adjust the pH to 6.8. This prepared the experimental conditions for the intestinal phase. Subsequently, 5 mL of simulated intestinal juice was added to the mixtures and further incubated for 8 h at 39 °C with shaking. The simulated intestinal juice was 0.2 mol/L phosphate buffer at pH 6.8, containing pancreatin (0.14 g/mL; P7545, Sigma-Aldrich, Saint Louis, USA), amyloglucosidase (1%; A7095, Sigma-Aldrich, Saint Louis, USA), and invertase (0.6 mg/mL; I4504, Sigma-Aldrich, Saint Louis, USA).

For determining the glucose release profiles, suspension samples (0.5 mL) were taken at different time points during the intestinal phase (0, 20, 60, 90, 120, 240, 360, and 480 min). For the determination of the nitrogen and amino acid releasing profiles, suspension samples (5 mL) were taken from the proportionally enlarged (by 2 times) digestion substrate and solution. And the sample collection time points included 60 and 120 min during the gastric phase, and 20, 60, 90, 120, 240, 360, and 480 min during the intestinal phase. All collected samples were immediately placed on ice to stop the digestion and then stored in a −20 °C refrigerator.

The glucose content of each sample was determined using a glucose oxidase kit (E1010, Applygen, Beijing, China). The determination of nitrogen concentrations of each sample was used method 954.01 from the Association of Official Analytical Chemists (AOAC) [22], and for total amino acid content using the micro amino acid content assay kit (BC1575, Solarbio, Beijing, China).

### The formula for calculating the nutrient release rate

The formula for calculating the release rate of glucose, soluble nitrogen, and total amino acids over specific intervals is as follows:

1 $$K = \frac{{D_{{t_{2} }} - D_{{t_{1} }} }}{{t_{2} - t_{1} }}$$

In this equation, *t* refers to the incubation time (min). *K* represents the release rate of glucose (g/100 g/min), or soluble nitrogen (g/kg/min), or total amino acids (mmol/kg/min). *D*_*t*_ is the total amount of nutrient released at *t* min.

### Diets

For this study, a nitrogen-free diet and 5 experimental diets were formulated, with the detailed composition provided in Table 1. As a marker, 0.3% chromic oxide was included in all diets. No protein feeds were added to the nitrogen-free diet. The experimental diets contained the same protein mix (casein, whey protein isolate, and isolated soybean protein, from Beijing Baishi Weixin Technology Co., Ltd., China), designed to represent common protein digestion rate of swine diets according to the data sets [9, 23, 24]. The starch in the nitrogen-free diet was sourced from common corn starch (from Beijing Baishi Weixin Technology Co., Ltd., China). Waxy corn starch (from Beijing Baishi Weixin Technology Co., Ltd., China) and high-amylose corn starch (from Xiangyu (Xinghua) Food Technology Co., Ltd., China) were used to design 5 experimental diets, intended to have varying rates and levels of glucose release. Briefly, the dietary treatments were as follows: 1) a diet that releases glucose rapidly and in large amounts (RGR_HGR), 2) a diet that releases glucose at the second fastest rate and in the second largest amounts (MRGR_MHGR), 3) a diet that releases glucose at a moderate rate and with moderate amounts of glucose release (MGR_MGR), 4) a diet that releases glucose at the second slowest rate and with the second lowest amounts of glucose release (MSGR_MLGR), 5) a diet that releases glucose slowly and with low amounts of glucose release (SGR_LGR), and 6) a diet free of nitrogen (NF) used exclusively for calculating standardized ileal amino acid digestibility in this study.

**Table 1:** Ingredients and nutrient composition of experimental diets (as-fed basis)^a^

Ingredients, %	RGR_HGR	MRGR_MHGR	MGR_MGR	MSGR_MLGR	SGR_LGR	NF
Normal maize starch	-	-	-	-	-	88.35
High amylose maize starch	-	17.30	34.60	51.90	69.20	-
Waxy maize starch	69.20	51.90	34.60	17.30	-	-
Isolated soybean protein	2.05	2.05	2.05	2.05	2.05	-
Casein	14.38	14.38	14.38	14.38	14.38	-
Whey protein isolate	2.72	2.72	2.72	2.72	2.72	-
Soybean oil	3.00	3.00	3.00	3.00	3.00	3.00
Cellulose acetate	4.00	4.00	4.00	4.00	4.00	4.00
Limestone	0.50	0.50	0.50	0.50	0.50	0.50
Dicalcium phosphate	2.50	2.50	2.50	2.50	2.50	2.50
Cr_2_O_3_	0.30	0.30	0.30	0.30	0.30	0.30
NaCl	0.45	0.45	0.45	0.45	0.45	0.45
K_2_CO_3_	0.30	0.30	0.30	0.30	0.30	0.30
MgO	0.10	0.10	0.10	0.10	0.10	0.10
Vitamin trace-mineral premix^b^	0.50	0.50	0.50	0.50	0.50	0.50
Analyzed nutrient levels						
Dry matter, %	97.72	97.91	97.81	97.74	98.13	97.69
Total starch, %	58.90	59.83	62.06	61.01	62.64	77.07
Amylopectin/Amylose ratio	16.45	3.69	1.65	0.76	0.35	3.58
Crude protein, %	9.51	9.47	9.54	9.48	9.59	-

^a^RGR_HGR, a diet that releases glucose rapidly and in large amounts; MRGR_MHGR, a diet that releases glucose at the second fastest rate and in the second largest amounts; MGR_MGR, a diet that releases glucose at a moderate rate and with moderate amounts of glucose release; MSGR_MLGR, a diet that releases glucose at the second slowest rate and with the second lowest amounts of glucose release; SGR_LGR, a diet that releases glucose slowly and with low amounts of glucose release; NF, a nitrogen free diet

^b^Vitamin trace-mineral premix provided the following per kg of complete diet for growing pigs: vitamin A, 5,512 IU; vitamin D_3_, 2,200 IU; vitamin E, 30 IU; vitamin K_3_, 2.2 mg; vitamin B_12_, 27.6 μg; riboflavin, 4.0 mg; pantothenic acid, 14.0 mg; niacin, 30.0 mg; choline chloride, 400.0 mg; folacin, 0.7 mg; thiamine 1.5 mg; pyridoxine 3.0 mg; biotin, 44.0 μg; Mn, 40.0 mg; Fe, 75.0 mg; Zn, 75.0 mg; Cu, 100.0 mg; I, 0.3 mg; Se, 0.3 mg

### The formulas for calculating the indicators reflecting the synchronization between dietary glucose release and nitrogen release

The indicators related to the synchronization between dietary glucose release and nitrogen release were proposed here. Two of them apply the relative change comparison principle to determine the extent of synchronization between the two nutrients released under different treatments. The formulas for calculating them are as follows:

2 $$Relative\;G/N=\frac{(K_\text{Glucose}/K_\text{GlucoseR})}{(K_\text{Nitrogen}/K_\text{NitrogenR})}$$

3 $$Relative\;G/AA=\frac{(K_\text{Glucose}/K_\text{GlucoseR})}{(K_\text{AA}/K_\text{AAR})}$$

In the two equations, *Relative G/N* represents the ratio of relative dietary glucose release to relative dietary soluble nitrogen release during the time interval. *Relative G/AA* represents the ratio of relative dietary glucose release to relative dietary total amino acids release during the time interval. When the ratio is greater than or equal to 1, the closer the ratio is to 1, the closer the relative change in nutrient release is, and the more they tend to be synchronized. *K*_Glucose_, *K*_Nitrogen_, and *K*_AA_ refer to the release rates of dietary glucose (g/kg/min), soluble nitrogen (g/kg/min), and total amino acids (mmol/kg/min), respectively, during the 0–20 min interval. *K*_GlucoseR_, *K*_NitrogenR_, and *K*_AAR_ refer to the averages of *K*_Glucose_, *K*_Nitrogen_, and *K*_AA_ across all diets, respectively.

The other two indicators help reveal the absolute ratios of nutrients release requirement. The formulas for calculating them are as follows:

4 $$G/N = \frac{{K_{{{\text{Glucose}}}} }}{{K_{{{\text{Nitrogen}}}} }}$$

5 $$G/AA = \frac{{K_{{{\text{Glucose}}}} }}{{K_{{{\text{AA}}}} }}$$

*G/N* represents the ratio of dietary glucose to dietary soluble nitrogen release during the time interval. *G/AA* represents the ratio of dietary glucose to dietary total amino acids release during the time interval. *K*_Glucose_, *K*_Nitrogen_, *K*_AA_, *K*_GlucoseR_, *K*_NitrogenR_, and *K*_AAR_ are as mentioned above.

### Animal housing and experimental design

Eighteen crossbred barrows (Duroc × Large White × Landrace) were surgically fitted with T-cannulas in the distal ileum. The surgical method followed the protocol described by Stein et al. [25]. All pigs (initial BW: 28.88 ± 3.06; 110 days old) were housed in individual metabolism cages (1.5 m × 1.2 m × 0.7 m) equipped with a nipple drinker and a feeder and were fed 2 percent (each meal) of their body weight at 8:00 and 16:00 with free access to water. For the duration of the experiment, the room temperature was kept at a constant range of 20 to 25 °C.

The experiment was conducted over two periods. In the first period, each diet was randomly assigned to 3 pigs for feeding. In the second period, to ensure the independence of the experiment, each diet was fed to a different set of three pigs that had not been previously exposed to that diet. Each period lasted for 9 d, comprising 5 d for diet adaptation, 2 d for feces and urine collection, and 2 d for ileal chyme collection. The weight of collected feces and volume of urine from each pig were recorded. All feces were collected and stored. After the daily urine sample was thoroughly mixed and filtered with gauze, a urine sample for each pig that did not exceed 100 mL in volume was collected. And the proportion of the collected urine volume to the total daily urine volume remained constant over the two days. The urine, feces, and ileal chyme intended for chemical analysis were all stored at −20 °C. The ileal chyme intended for microbiota analysis was stored at −80 °C. At the end of each period, samples collected from each pig were pooled. Then feces and chyme underwent lyophilization for a duration of 72 h in a freeze dryer. At the end of each trial period, the body weight and feed consumption of each pig were also recorded.

### Chemical analyses of feed ingredients, diets, chyme, feces, and urine samples

The total starch level of feed ingredients was detected using the previously described method [26, 27]. The total starch level of each diet was determined using a starch assay kit (K-TSTA-100A, Megazyme, Bray, Ireland). Amylose/amylopectin ratio in each diet was assessed and analyzed employing a polysaccharide test kit (K-AMYL, Megazyme, Bray, Ireland). The dry matter (DM) of feed ingredients, diets, chyme, and feces was analyzed, and the crude protein of diets, chyme, and feces was quantified, both according to the procedures descried by AOAC [22]. The gross energy (GE) of diets and feces was measured in accordance with the international benchmark ISO 9831:1998 [28], employing an oxygen bomb calorimeter (model 6400, Parr Corporation, USA). The level of chromium for diets and chyme was detected following the procedure detailed by Fenton [29].

The quantification of amino acids in the diets and chyme was conducted in accordance [30]. Briefly, samples were hydrolyzed with 6 mol/L HCl at 110 °C for 24 h and then analyzed for 15 amino acids using an automatic amino acid analyzer (Hitachi L-8900, Tokyo, Japan). For the determination of methionine and cystine, samples were first subjected to cold performic acid oxidation, then hydrolyzed with 7.5 mol/L HCl at 110 °C for 24 h, and finally analyzed using an amino acid analyzer (Hitachi L-8800, Tokyo, Japan). Tryptophan was determined after the sample was hydrolyzed with LiOH at 110 °C for 22 h using a high-performance liquid chromatography system (Agilent 1200, Agilent, Palo Alto, USA). Analyzed chemical composition of feed ingredients and different diets were shown in Table S1 and Table S2.

### Definitions and equations for apparent nutrient and energy digestibility

The ATTD of energy and nitrogen of each diet were calculated as follows:

6 $$ATTD_\text{nutrient}=(FI_\text{nutrient}-Feces_\text{nutrient})/FI_\text{nutrient}\times100$$

*ATTD*_nutrient_ is the apparent total tract digestibility of nitrogen or energy (%). *FI*_nutrient_ is the amounts of nitrogen (g) or energy (MJ/kg) ingested. *Feces*_nutrient_ is amounts of energy (MJ/kg) or nitrogen (g) voided via feces.

The equation used to calculate the AID of amino acids of each diet was according to the equation described by Stein et al. [31].

7 $$AID=\left[1-(AA_\text{digesta}/AA_\text{diet})\times(C{\text{r}}_\text{diet}/C{\text{r}}_\text{digesta}\text{)}\right]\times100$$

8 $$IAA = AA_{{{\text{digesta}}}} \times \left( {Cr_{{{\text{diet}}}} /Cr_{{{\text{digesta}}}} } \right)$$

 

9 $$SID = AID + \left( {IAA/AA_{{{\text{diet}}}} } \right) \times 100$$

*AID* is the apparent ileal digestibility of amino acid (%). *AA*_digeseta_ and *AA*_diet_ represent the amino acid concentration (g/kg of DM) in ileal chyme and diet, respectively. *Cr*_diet_ and *Cr*_digesta_ are the chromium concentration (g/kg of DM) in the diet and ileal chyme, respectively. *IAA* is the basal ileal endogenous loss of an amino acid (g/kg of DM intake), measured in the nitrogen free diet. *SID* is the standardized ileal digestibility of amino acids (%).

### Characterizing molecular size distributions during digestion

Protein was extracted from ileal chyme using the Total Protein Extraction Kit (EX1101, Solarbio, Beijing, China). The extracted protein was then quantified using the Protein Quantification Kit (KTD3001, Abbkine, Wuhan, China). According to this, extracted samples were diluted to the same protein concentration, and 500 μL of ultrapure water was added to each. Before being analyzed, the solutions were resuspended using 15 s of sonication [32]. Size exclusion-high performance liquid chromatography (SE-HPLC) analysis was performed using an Agilent 1200 HPLC system equipped with a 300 mm × 7.8 mm BioCore SEC-300 column (NanoChrom, Suzhou, China). Isocratic runs were carried out with a mobile phase of 50 mmol/L phosphate buffer solution containing 300 mmol/L sodium chloride (pH 6.8) and a flow rate of 0.5 mL/min, while maintaining a column temperature of 25 °C. Eluted components were detected by ultraviolet absorption at 220 nm. The aforementioned method was developed based on the guidelines provided by NanoChrom for the column, and referenced the procedure detailed by Liu et al. [33].

### 16S rRNA sequencing and data analysis

DNA extraction from ileal chyme was performed using the FastPure Stool DNA Isolation Kit (MJYH, Shanghai, China). The V3–V4 regions of the bacterial 16S rRNA gene were amplified using the primers 338F (5′-ACTCCTACGGGAGGCAGCAG-3′) and 806R (5′-GGACTACHVGGGTWTCTAAT-3′) on an ABI GeneAmp 9700 PCR thermocycler (ABI, Foster City, USA). The sequencing process was carried out on the Illumina NextSeq 2000 PE300 platform (Illumina, San Diego, USA) following the standard protocols provided by Majorbio Bio-Pharm Technology Co., Ltd. (Shanghai, China). Following demultiplexing, the obtained sequences were quality-filtered using fastp (v0.19.6) [34] and subsequently merged using FLASH (v1.2.11) [35]. Sequences were then imported into QIIME2 (version 2020.2) and DADA2 was used with default parameters for denoising, generating Amplicon Sequence Variants (ASVs). The taxonomic assignment of ASVs was carried out using the Naive Bayes consensus taxonomy classifier available in Qiime2, in conjunction with the SILVA 16S rRNA database (v138). To mitigate the bias arising from disparities in sequencing depth, all samples were rarefied to 27,081 sequences, with average Good’s coverage reaching 99.95%.

The diversity indices, including α-diversity and β-diversity, were computed utilizing the vegan package (version 2.6-8) [36]. Kruskal-Wallis test was used to compare α-diversity among treatments. PCoA was performed based on the weighted Bray-Curtis distance. PERMANOVA was conducted using the adonis function from the ‘vegan’ package (with 999 permutations) to evaluate the factors shaping microbiota. Visualization of the ASVs’ network across all samples was achieved using the igraph (version 2.0.3) R package and Gephi [37]. During this procedure, ASVs with a relative abundance above the filter threshold of 0.0001, a Spearman’s correlation coefficient greater than 0.6 with other ASVs, and a corresponding *P*-value less than 0.05 were selected. Fast greedy algorithm was used to partition the modules. To identify the differential modules according to the module eigengene, Kruskal-Wallis test and Wilcoxon rank-sum test were used. Wilcoxon rank-sum test was also used to compare the relative abundance of ASVs between groups.

Functional prediction of the ileal chyme microbiota was conducted using two complementary approaches. FAPROTAX [38], which is suitable for predicting biogeochemical cycling in environmental samples with a focus on carbon and nitrogen cycling, was used here to investigate the microbiota related to carbon and nitrogen utilization. Then, due to a limitation of FAPROTAX, which is based on validated literature on cultivable bacteria and may have lower prediction coverage, PICRUSt2 was also employed [39]. Kruskal-Wallis test and Welch’s *t*-test were conducted to identify differences in the proportions of microorganisms involved in different functions between treatments, with FDR adjustment applied to the *P* values. The analysis method was partly referenced by previous studies [40, 41].

### Statistical analysis of data excluding 16S rRNA data

ANOVA was applied to analyze short-term growth performance, energy and nitrogen utilization data, as well as data on the synchronization of nutrient release, and the release rate and amount of glucose, soluble nitrogen, and total amino acids from feed ingredients or diets. To separate the means of treatments, the *t*-test or Tukey’s post-hoc test was employed. Results are presented as means ± SEM. For identifying differences in amino acid digestibility among groups, Kruskal-Wallis test and Wilcoxon rank-sum test were used. To evaluate the relationships among various nutrient release rates, a Pearson correlation analysis was carried out. All statistical analyses were conducted using R software (version 4.4.2).

## Results

### Determination of the in vitro glucose release patterns including release rates and amounts for different feed ingredients

**Fig. 1:**
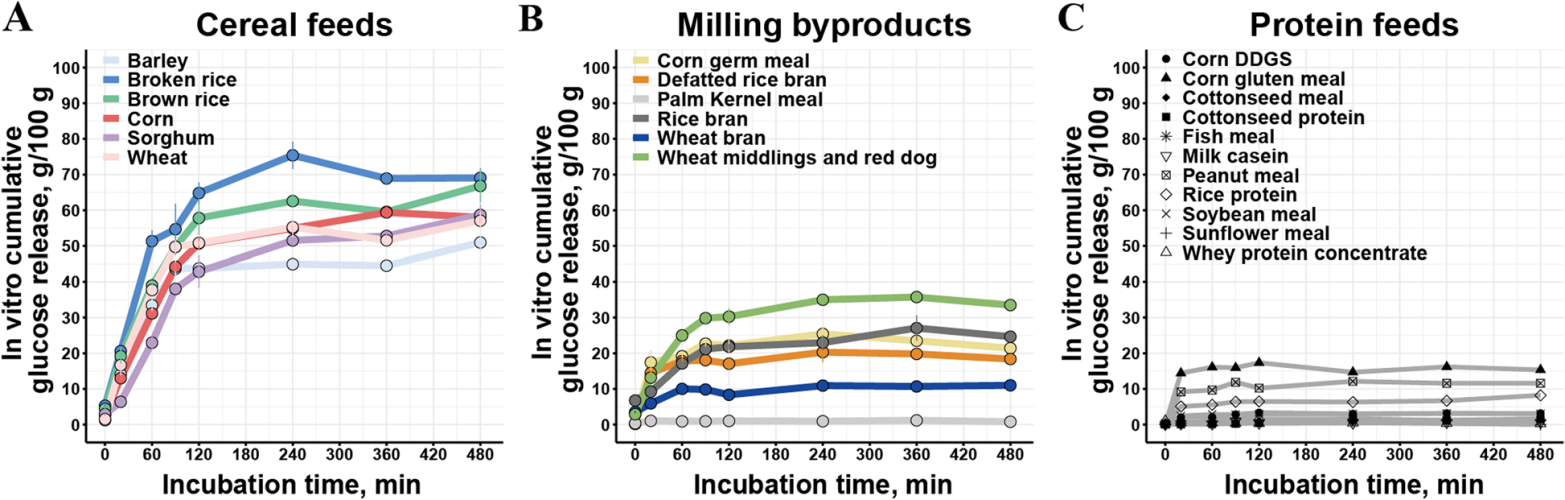
Time course of the average cumulative in vitro glucose release from feed ingredients during the simulated intestinal phase. **A** Glucose release of cereal feeds. **B** Glucose release of milling byproducts. **C** Glucose release of protein feeds. *n* = 3 for each feed ingredient

By comparing the total amounts of glucose released at various time points for all feed ingredients, significant differences were found among the different feed ingredients (*P* < 0.05). Sorghum, rice bran, wheat bran, palm kernel meal and most protein feeds released glucose slowly during the initial digestion phase (0–20 min), whereas broken rice, brown rice, wheat, and corn germ meal showed a faster glucose release (Fig. 1). Although barley, corn germ meal, defatted rice bran, corn gluten meal and wheat middlings and red dog initially exhibited a slightly higher glucose release rate compared to corn (Fig. 1B and C), corn demonstrated a greater overall glucose release throughout the entire incubation period (Fig. 1A). Considering the glucose release patterns of feed ingredients, a distinction can be made based on their rate of release (slowly, rapidly), the level of glucose released (high, low), and the duration of release (persistent, transient).

### Rapid release of glucose in vitro contributed to the synchronized release of glucose and nitrogen in diets

**Fig. 2:**
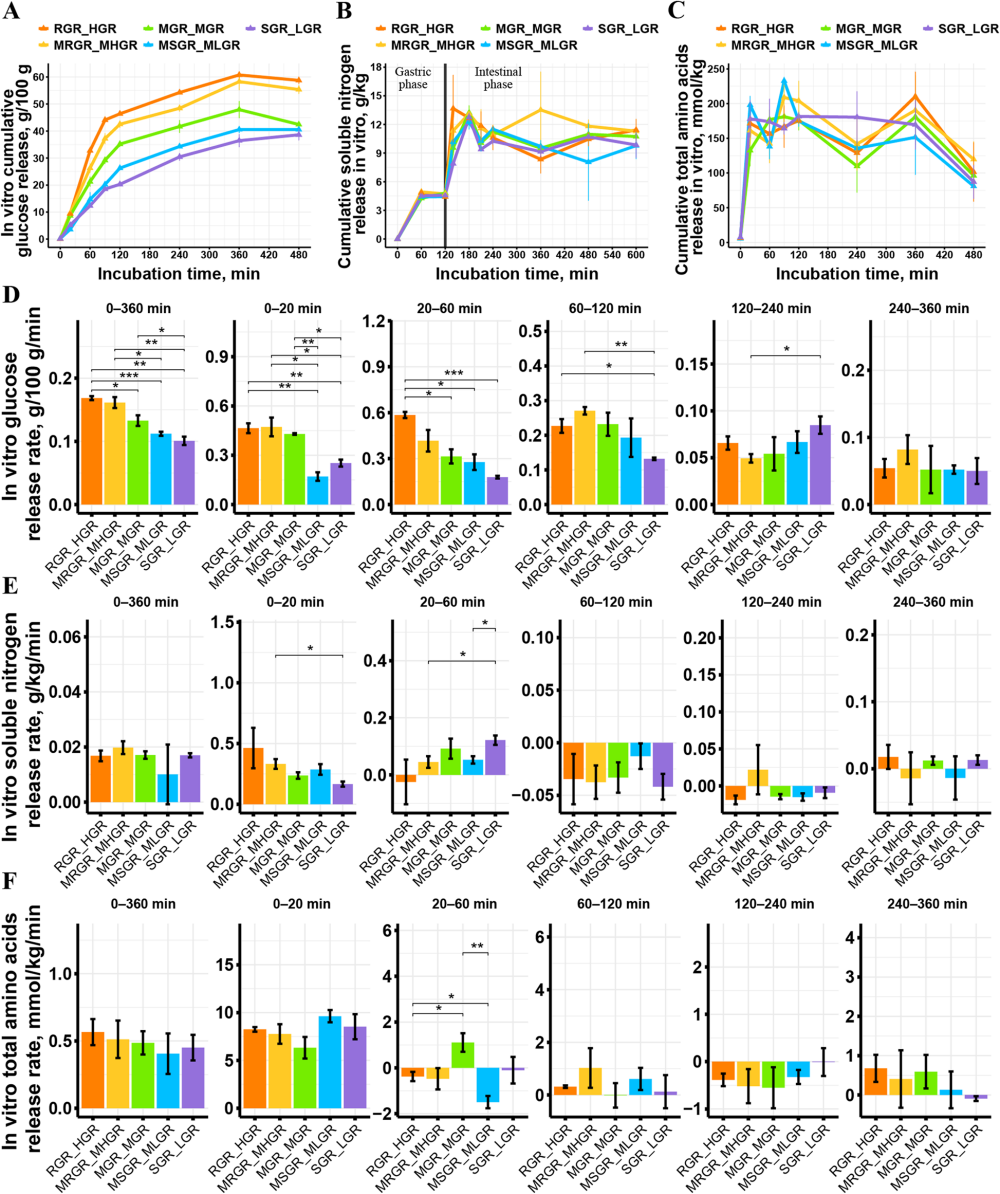
Time course of the average in vitro nutrient release from diets. **A** Cumulative glucose release of diets during the simulated intestinal digestion phase in vitro. **B** Cumulative soluble nitrogen release of diets during the simulated gastric and intestinal digestion phase in vitro. **C** Cumulative total amino acids release of diets during the simulated intestinal digestion phase in vitro. **D** The glucose release rate of diets in different digesting intervals (simulated intestinal phase). **E** The soluble nitrogen release rate of diets in different digesting intervals (simulated intestinal phase). **F** The total amino acids release rate of diets in different digesting intervals (simulated intestinal phase). RGR_HGR, a diet that releases glucose rapidly and in large amounts; MRGR_MHGR, a diet that releases glucose at the second fastest rate and in the second largest amounts; MGR_MGR, a diet that releases glucose at a moderate rate and with moderate amounts of glucose release; MSGR_MLGR, a diet that releases glucose at the second slowest rate and with the second lowest amounts of glucose release; SGR_LGR, a diet that releases glucose slowly and with low amounts of glucose release; * indicates *P* < 0.05, ** indicates *P* < 0.01, and *** indicates *P* < 0.001. No marking indicates there is no significance. *n* = 3 for each diet

Five diets were designed to investigate the role of glucose release characteristics of feed ingredients in synchronizing dietary carbon and nitrogen supply. As shown in Fig. 2A and D, all diets released glucose primarily within the first 120 min of the simulated intestinal digestion phase, with a rapid release in the first 60 min. During the stage when diets released glucose rapidly, dietary soluble nitrogen was also released rapidly, specifically during the 0–20 min period (Fig. 2B and E). This was the stage of rapid release for dietary amino acids as well (Fig. 2C and F). Furthermore, there was a weak positive correlation between the release rate of dietary soluble nitrogen and glucose in this stage (Fig. S1A). However, the total amino acid release rate of different diets did not follow this rhythm (Fig. S1B). It was not until the late stage of the simulated intestinal digestion phase that a positive correlation was observed between the total amino acid release rate and the glucose release rate. The variation in nitrogen supply patterns among diets and these correlations indicated that the rate of dietary glucose release not only directly altered the pattern of carbon supply but also influenced the rhythm of nitrogen supply, resulting in different diets exhibiting distinct patterns of carbon and nitrogen supply.

Table 2 presents the level of synchronization between glucose and nitrogen release from diets during a common stage of rapid nutrient release (the 0–20 min simulated intestinal digestion stage). Although the *Relative G/N* and *Relative G/AA* of the RGR_HGR and MRGR_MHGR were not equal to 1, the parameters of these two diets were closer to 1 than those of the other diets, suggesting better synchronization of glucose and nitrogen release. While diets with lower glucose release rate exhibited asynchronous carbon and nitrogen supply. As shown in Fig. 2, MSGR_MLGR and SGR_LGR had a high rate of total amino acids release while released glucose slowly. The *Relative G/AA* of these two diets was much lower than 1 (Table 2), indicating an asynchronous pattern of dietary carbon and nitrogen release, with insufficient glucose supply and relatively excessive nitrogen release. In contrast, MGR_MGR exhibited another asynchronous pattern of dietary carbon and nitrogen release, characterized by relatively excessive glucose release and insufficient nitrogen supply. Both the *Relative G/N* and *Relative G/AA* values for MGR_MGR were higher than 1, and these values were also higher than those of the other diets (Table 2). According to the results above, RGR_HGR and MRGR_MHGR were classified as diets with better synchronous release of dietary glucose and nitrogen (Syn), while MGR_MGR, MSGR_MLGR, and SGR_LGR were classified as diets with asynchronous release of dietary glucose and nitrogen (Asyn).

**Table 2:** Synchronization of dietary glucose, soluble nitrogen, and total amino acid release^1,2^

Diets	Relative G/N	Relative G/AA	G/N	G/AA, g/mmol
RGR_HGR	1.23 ± 0.59	1.28 ± 0.12^ab^	14.80 ± 7.21	0.57 ± 0.05^ab^
MRGR_MHGR	1.22 ± 0.23	1.40 ± 0.15^ab^	14.70 ± 2.83	0.62 ± 0.07^ab^
MGR_MGR	1.54 ± 0.17	1.63 ± 0.26^a^	18.60 ± 2.13	0.72 ± 0.12^a^
MSGR_MLGR	0.53 ± 0.14	0.41 ± 0.07^c^	6.41 ± 1.69	0.18 ± 0.03^c^
SGR_LGR	1.32 ± 0.24	0.71 ± 0.14^b^	16.00 ± 2.94	0.31 ± 0.06^b^

^1^RGR_HGR, a diet that releases glucose rapidly and in large amounts; MRGR_MHGR, a diet that releases glucose at the second fastest rate and in the second largest amounts; MGR_MGR, a diet that releases glucose at a moderate rate and with moderate amounts of glucose release; MSGR_MLGR, a diet that releases glucose at the second slowest rate and with the second lowest amounts of glucose release; SGR_LGR, a diet that releases glucose slowly and with low amounts of glucose release

^2^*Relative G/N* represents the ratio of relative dietary glucose release to relative dietary soluble nitrogen release. *Relative G/AA* represents the ratio of relative dietary glucose release to relative dietary total amino acids release. *G/N* represents the ratio of dietary glucose to dietary soluble nitrogen release. *G/AA* represents the ratio of dietary glucose to dietary total amino acids release. These ratios were calculated at 0–20 min (simulated intestinal digestion phase). When the *Relative G/N* or *Relative G/AA* is greater than or equal to 1, the closer the ratio is to 1, the closer the relative change in nutrient release is, and the more they tend to be synchronized

^a,b,c^In the same column followed by different letters for *Relative G/AA* and *G/AA* differ by Tukey’s post-hoc test (*P* < 0.05). *n* = 3 for each diet. All values are expressed as mean ± SEM

### Synchronized release of glucose and nitrogen from diets promoted short-term daily gain in growing pigs

The short-term growth performance of pigs fed Syn diet and Asyn diet was compared, as shown in Table 3. Lower weight gain was observed in the pigs fed diets with asynchronous release of carbon and nitrogen release (*P* < 0.05). And pigs fed the MRGR_MHGR diet had the greatest average daily gain (ADG) among groups. However, there was no significant difference in average daily feed intake between treatment groups.

**Table 3:** Effect of diets with differences in synchronization of glucose and nitrogen release on short-term growth performance and energy and nitrogen utilization of pigs^1,2^

Items	Syn	Asyn	Diets					*P*_*1*_	*P*_*2*_
			RGR_HGR	MRGR_MHGR	MGR_MGR	MSGR_MLGR	SGR_LGR		
ADG, kg/d	0.58 ± 0.03^a^	0.47 ± 0.02^b^	0.56 ± 0.04^ab^	0.61 ± 0.04^a^	0.48 ± 0.06^ab^	0.49 ± 0.02^ab^	0.44 ± 0.04^b^	< 0.01	0.05
ADFI, kg/d	1.24 ± 0.04	1.26 ± 0.03	1.26 ± 0.05	1.22 ± 0.06	1.25 ± 0.06	1.24 ± 0.02	1.28 ± 0.07	0.74	0.96
ATTDGE, %	63.30 ± 2.26^a^	56.20 ± 2.13^b^	60.10 ± 3.59	66.40 ± 2.35	56.80 ± 3.11	55.30 ± 3.62	56.40 ± 4.85	0.03	0.21
FE/FIGE, %	36.70 ± 2.26^b^	43.80 ± 2.13^a^	39.90 ± 3.59	33.60 ± 2.35	43.20 ± 3.11	44.70 ± 3.62	43.60 ± 4.85	0.03	0.21
UE/FIGE, %	1.88 ± 0.17^a^	1.83 ± 0.22^b^	1.82 ± 0.21	1.95 ± 0.28	1.81 ± 0.33	2.32 ± 0.49	1.37 ± 0.24	0.88	0.37
FN/FIN, %	64.20 ± 3.84^y^	77.20 ± 5.03^x^	71.20 ± 2.90^xy^	58.50 ± 5.81^y^	79.20 ± 4.31^xy^	86.50 ± 10.00^x^	65.90 ± 9.76^xy^	0.08	0.09
UN/FIN, %	14.50 ± 1.11	12.30 ± 1.10	13.80 ± 1.24	15.10 ± 1.83	15.20 ± 1.17	12.30 ± 1.68	9.32 ± 2.14	0.19	0.10

^1^Syn, diets with better synchronous release of dietary glucose and nitrogen (RGR_HGR, MRGR_MHGR); Asyn, diets with asynchronous release of dietary glucose and nitrogen (MGR_MGR, MSGR_MLGR, SGR_LGR); RGR_HGR, a diet that releases glucose rapidly and in large amounts; MRGR_MHGR, a diet that releases glucose at the second fastest rate and in the second largest amounts; MGR_MGR, a diet that releases glucose at a moderate rate and with moderate amounts of glucose release; MSGR_MLGR, a diet that releases glucose at the second slowest rate and with the second lowest amounts of glucose release; SGR_LGR, a diet that releases glucose slowly and with low amounts of glucose release

^2^ADG, average daily gain; ADFI, average daily feed intake; ATTDGE, the apparent total tract digestibility of gross energy; FE/FIGE, the ratio of fecal energy to energy intake; UE/FIGE, the ratio of urinary energy to energy intake; FN/FIN, the ratio of fecal nitrogen to nitrogen intake; UN/FIN, the ratio of urinary nitrogen to nitrogen intake

^a,b^In the same row followed by different letters for items differ by the independent sample* t*-test or Tukey’s post-hoc test (*P* < 0.05); ^x,y^In the same row followed by different letters for items differ by the independent sample t test or Tukey’s post-hoc test (0.05 < *P* < 0.10); *P*_1_, the *P* value of t test between the Asyn, Syn group; *P*_2_, the *P* value of ANOVA among the RGR_HGR, MRGR_MHGR, MGR_MGR, MSGR_MLGR, SGR_LGR diets. *n* = 6 for each diet. All values are expressed as mean ± SEM

### Synchronized release of glucose and nitrogen of diets reduced the energy and nitrogen losses

As shown in Table 3, the apparent total tract digestibility of gross energy was greater in pigs fed Syn diet (*P* < 0.05). Less energy excretion was observed in pigs fed Syn diet (*P* < 0.05), especially in those fed MRGR_MHGR diet (*P* < 0.05). Moreover, the fecal nitrogen excretion also tended to be lower in pigs fed Syn diet (0.05 < *P* < 0.10). Fecal nitrogen excretion tended to be lower in MRGR_MHGR compared to MSGR_MLGR (0.05 < *P* < 0.10). However, no notable difference was observed in the urinary excretion of energy and nitrogen across the different treatments.

### Asynchronous release of dietary glucose and nitrogen induced a pronounced shift in the digestibility of nitrogenous nutrients in the terminal ileum of pigs

**Fig. 3:**
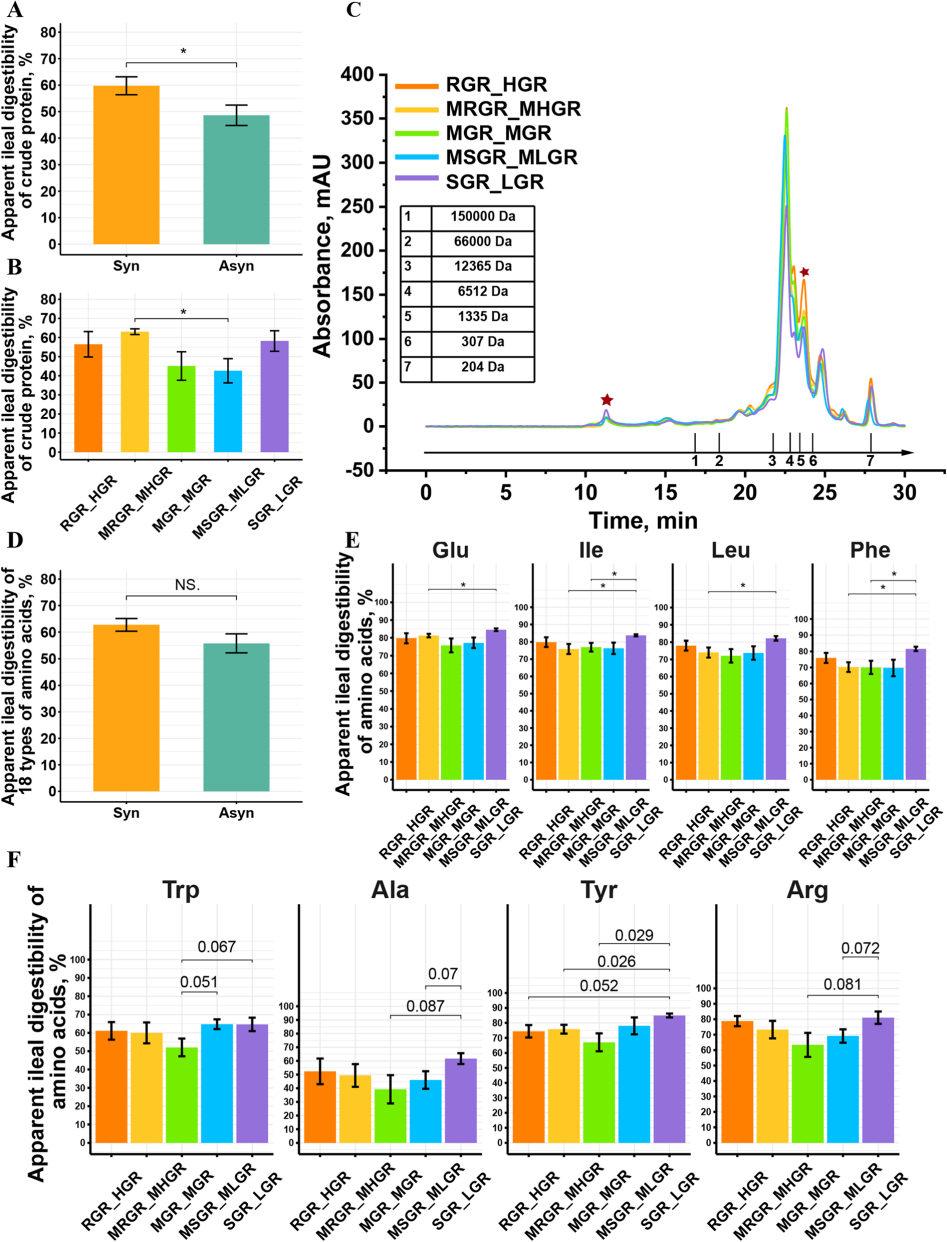
Asynchronous release of dietary glucose and nitrogen induced a pronounced shift in the digestibility of nitrogenous nutrients in the terminal ileum of pigs. **A** Differences in the apparent ileal digestibility of crude protein between Syn and Asyn group. **B** Differences in the apparent ileal digestibility of crude protein among treatments. **C** The molecular size distributions of the protein of ileal chyme. The first red star highlights the peak time of the first obvious large molecular size. The second red star indicates the peak time of the approximate molecular size of oligopeptides. **D** Differences in the apparent ileal digestibility of 18 amino acids between Syn and Asyn group. **E** and **F** Differences in the apparent ileal digestibility of each amino acid among treatments. RGR_HGR, a diet that releases glucose rapidly and in large amounts; MRGR_MHGR, a diet that releases glucose at the second fastest rate and in the second largest amounts; MGR_MGR, a diet that releases glucose at a moderate rate and with moderate amounts of glucose release; MSGR_MLGR, a diet that releases glucose at the second slowest rate and with the second lowest amounts of glucose release; SGR_LGR, a diet that releases glucose slowly and with low amounts of glucose release; Syn, diets with better synchronous release of dietary glucose and nitrogen (RGR_HGR, MRGR_MHGR); Asyn, diets with asynchronous release of dietary glucose and nitrogen (MGR_MGR, MSGR_MLGR, SGR_LGR); * indicates *P* < 0.05, NS. indicates there are no significance. *n* = 6 for each diet

As shown in Fig. 3A, pigs fed the Syn diet exhibited significantly higher apparent ileal digestibility (AID) of crude protein (*P* < 0.05). Among all treatments, the MRGR_MHGR group had the highest AID of crude protein (Fig. 3B). Notably, there was no significant difference in the AID of crude protein between pigs fed the SGR_LGR diet and those fed the RGR_HGR or MRGR_MHGR diets. However, the protein extracted from the ileal chyme of pigs fed the SGR_LGR diet showed a higher proportion of large molecular weight particles (> 150 kDa) compared to the others, while it exhibited a lower proportion of small molecular weight particles ranging from 307 to 1,335 Da (Fig. 3C).

The apparent ileal digestibility of amino acids among the different treatments is shown in Fig. 3D–F, Table S3, and Table S4. The AID of total amino acids was higher in pigs fed Syn diets compared to those fed Asyn diets (Fig. 3D). However, the AID of different amino acids exhibited varying trends in pigs fed Asyn diets with different characteristic (Fig. 3E and F). There were no significant differences in the AID of cysteine, valine, and methionine among pigs fed the MSGR_MLGR diet, SGR_LGR diet, MRGR_MHGR diet, and RGR_HGR diet (Table S3). The AID of glutamate, isoleucine, leucine, and phenylalanine was significantly higher in pigs fed the SGR_LGR diet compared to those fed the MRGR_MHGR diet (*P* < 0.05, Fig. 3E). While compared to the MSGR_MLGR and SGR_LGR diets, pigs fed the MGR_MGR diet tended to have a lower AID of tryptophan (0.05 < *P* < 0.10, Fig. 3F). Additionally, the AID of tyrosine was lower, while alanine, and arginine tended to be lower in pigs fed the MGR_MGR diet compared to those fed the SGR_LGR diet (*P* < 0.10). It was observed that the pigs fed the MGR_MGR diet had lower AID of most amino acids compared to the other treatments (Table S3). The differences in the SID among pigs fed different diets were comparable to the differences in the AID among pigs fed different diets, as indicated in the Table S4.

### Asynchronous release of dietary glucose and nitrogen showed distinct microbial signatures in the terminal ileum of pigs

**Fig. 4:**
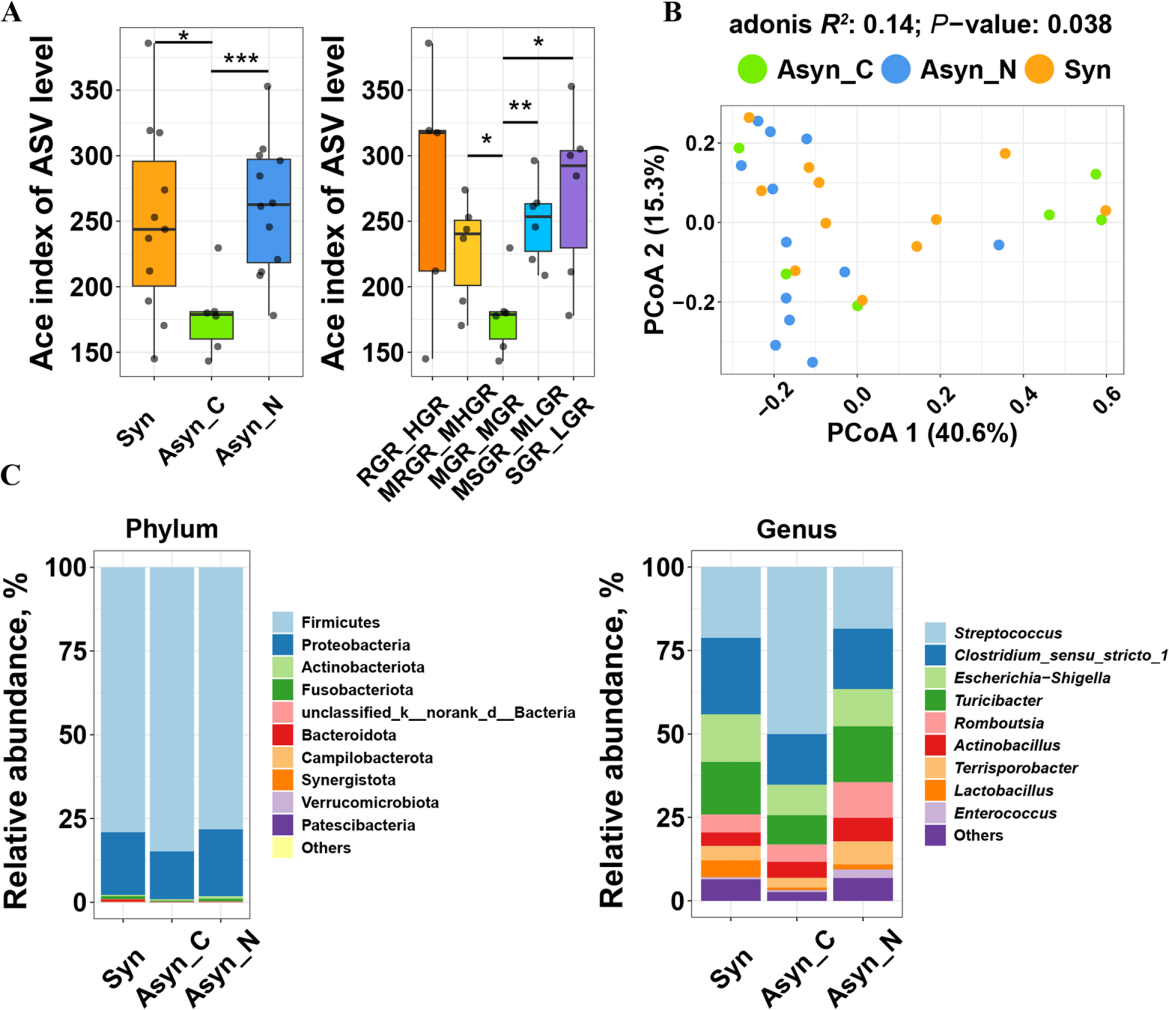
Response of ileal microbiome composition to asynchronous release of dietary glucose and nitrogen. **A** Boxplots of alpha diversity as measured by Abundance-based coverage estimator (ACE) of the ileal microbiome. **B** PCoA of the ileal microbiome based on the weighted Bray-Curtis distances metric. **C** Relative abundance of bacteria at phylum and genus level. RGR_HGR, a diet that releases glucose rapidly and in large amounts; MRGR_MHGR, a diet that releases glucose at the second fastest rate and in the second largest amounts; MGR_MGR, a diet that releases glucose at a moderate rate and with moderate amounts of glucose release; MSGR_MLGR, a diet that releases glucose at the second slowest rate and with the second lowest amounts of glucose release; SGR_LGR, a diet that releases glucose slowly and with low amounts of glucose release; Syn, diets with better synchronous release of dietary glucose and nitrogen (RGR_HGR, MRGR_MHGR); Asyn, diets with asynchronous release of dietary glucose and nitrogen (Asyn_C: MGR_MGR, Asyn_N: MSGR_MLGR, SGR_LGR); *n* = 6 for each diet except RGR_HGR, where *n* = 5. * indicates *P* < 0.05, ** indicates *P* < 0.01, and *** indicates *P* < 0.001. No marking indicates there is no significance

No significant difference was observed in the α-diversity of ileal chyme, as measured by the Abundance-based coverage estimator (ACE) metric, between pigs fed the Syn diet and those fed the Asyn diet (Fig. S2A). However, there were significant differences in the ACE of ileal chyme among pigs fed Asyn diets with different characteristics (Fig. 4A). Specifically, the ACE of ileal chyme of pigs fed the MGR_MGR diet was lower than that of pigs fed the MSGR_MLGR and SGR_LGR diets (*P* < 0.05). No significant community shift was observed in chyme between pigs fed the Syn diet and Asyn diet (Adonis, *P* = 0.17, Fig. S2B) or among different diets (Adonis, *P* = 0.16, Fig. S2B). Despite this, adonis analysis indicated significant differences among Syn (RGR_HGR, MRGR_MHGR), Asyn_C (MGR_MGR), and Asyn_N (MSGR_MLGR, SGR_LGR) treatments based on Bray-Curtis distances (Adonis, *R*^2^ = 0.14, *P* < 0.05, Fig. 4B). At the phylum level, the microbiota was dominated by Firmicutes and Proteobacteria in Syn, Asyn_C, and Asyn_N treatments (Fig. 4C). At the genus level, the microbiota was dominated by *Streptococcus* and *Clostridium_sensu_stricto_1*, followed by *Turicibacter* and *Escherichia-Shigella* in these treatments (Fig. 4C).

**Fig. 5:**
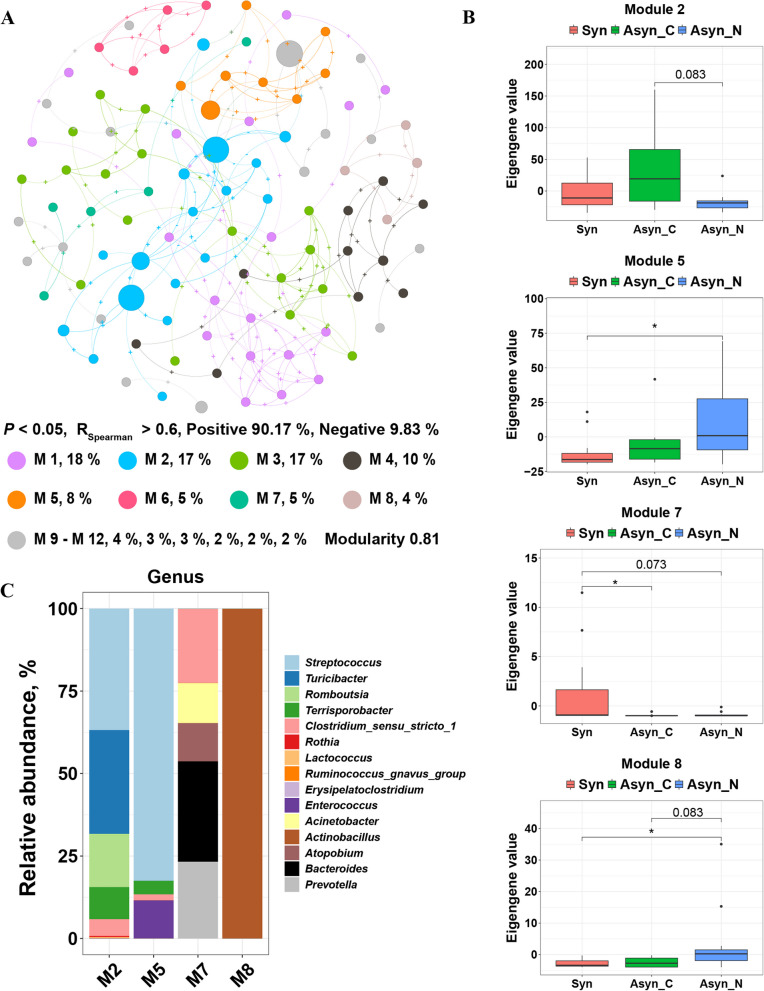
Bacterial co-occurrence networks and the divided modules. **A** Co-occurrence network analysis was based on ASVs from all samples (*r* > 0.6, *P* < 0.05, Spearman correlation, *n* = 29). ASVs are colored according to their assigned modules. The size of each node represents the relative abundance of the corresponding ASV. **B** Modules showed differences among the Syn, Asyn_C, and Asyn_N treatments. * indicates *P* < 0.05, no marking indicates there is no significance. **C** Relative abundance of module members at genus level. RGR_HGR, a diet that releases glucose rapidly and in large amounts; MRGR_MHGR, a diet that releases glucose at the second fastest rate and in the second largest amounts; MGR_MGR, a diet that releases glucose at a moderate rate and with moderate amounts of glucose release; MSGR_MLGR, a diet that releases glucose at the second slowest rate and with the second lowest amounts of glucose release; SGR_LGR, a diet that releases glucose slowly and with low amounts of glucose release; Syn, diets with better synchronous release of dietary glucose and nitrogen (RGR_HGR, MRGR_MHGR); Asyn, diets with asynchronous release of dietary glucose and nitrogen (Asyn_C: MGR_MGR; Asyn_N: MSGR_MLGR, SGR_LGR); *n* = 6 for each diet except RGR_HGR, where *n* = 5

The co-occurrence network comprised 100 nodes and 173 edges, with 90.17% of the edges being positively correlated and 9.83% being negatively correlated, which showed the interactive relationships among bacteria (Fig. 5A). The microorganisms were clustered into 14 modules. Four of these modules demonstrated differences among the Syn, Asyn_C, and Asyn_N treatments based on the module eigengenes (Fig. 5B). Module 2 contained more members than the other three modules (Fig. 5A). Its module eigengene tended to be higher in Asyn_C compared to that in Asyn_N (0.05 < *P* < 0.10, Fig. 5B). At the genus level, the members of Module 2 were dominated by *Streptococcus* and *Turicibacter*. The difference between the Asyn_N and Syn was highlighted in Module 5 and Module 8. As shown in Fig. 5C, the microbiota was dominated by *Streptococcus* and *Enterococcus* in Module 5. And all members of Module 8 were annotated as *Actinobacillus* at the genus level. Moreover, Module 7 showed the differences between Syn and Asyn treatments. At the genus level, the microbiota was dominated by *Bacteroides*, *Prevotella*, and *Clostridium_sensu_stricto_1* in this module. However, the expression for Module 7 was much lower than that for the other modules (Fig. 5A and B). And there was no significant difference in the relative abundance of *Bacteroides* in Module 7 between treatments (Fig. S3).

**Fig. 6:**
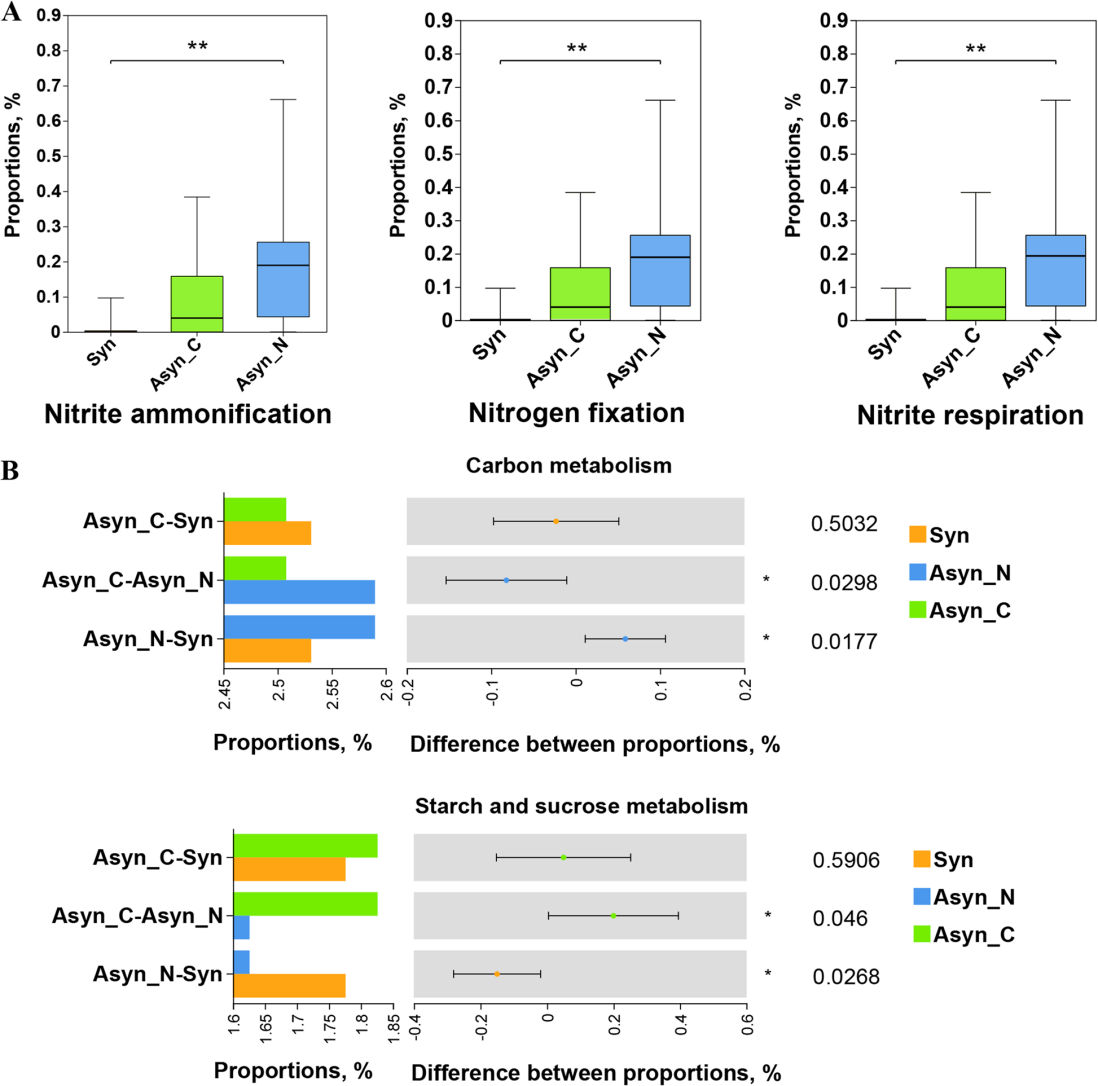
Microbial functional analysis. **A** FAPROTAX analysis showed the proportion of microorganisms involved in N cycling processes varied significantly among treatments. **B** PICRUSt2 functional prediction showed that Asyn diets altered carbon metabolism. Syn, diets with better synchronous release of dietary glucose and nitrogen (RGR_HGR, MRGR_MHGR); Asyn, diets with asynchronous release of dietary glucose and nitrogen (Asyn_C: MGR_MGR; Asyn_N: MSGR_MLGR, SGR_LGR); *n* = 6 for each diet except RGR_HGR, where *n* = 5. * indicates *P* < 0.05, ** indicates *P* < 0.01, no marking indicates there is no significance

To investigate the variation in functional microbial communities among Syn, Asyn_C, and Asyn_N treatments, FAPROTAX analysis was conducted. ASVs in ileal chyme samples were annotated and linked to 36 pathways (Table S5). Specifically, the N cycling processes varied significantly among groups (Fig. 6A). The Asyn_N group had a significantly higher proportion of microorganisms involved in nitrite ammonification, nitrogen fixation, and nitrite respiration compared to the Syn group (*P* < 0.01). The proportion of microbial communities involved in nitrite ammonification, nitrogen fixation, and nitrite respiration at SGR_LGR was significantly higher than that at RGR_HGR and MRGR_MHGR (Fig. S4, *P* < 0.01). Additionally, PICRUSt2 functional prediction showed that Asyn diets altered carbon metabolism (Fig. 6B). The Asyn_N group exhibited a significantly higher proportion of microorganisms associated with carbon metabolism than the remaining groups (Fig. 6B, *P* < 0.05). Conversely, the proportion of microorganisms associated with starch and sucrose metabolism significantly decreased in the Asyn_N groups (Fig. 6B, *P* < 0.05).

## Discussion

Determining the glucose release kinetics of different feed ingredients in vitro helps us follow the digestive processes within the digestive tract in more detail [42, 43]. For instance, broken rice and wheat showed faster and higher total glucose release throughout digestion, while sorghum exhibited a lower release rate in the initial stage. This is in agreement with the results observed in earlier studies [43–45]. And our extended investigation into the in vitro glucose release patterns of non-cereal feeds or protein feeds innovatively revealed that there were also significant differences among low-starch feed ingredients. Together, these findings indicated that significant variations existed in the release patterns of glucose among different feed ingredients during digestion. And it may correlate with various factors such as the source, the ratio of amylopectin to amylose, and the particle size of the starch [46].

What implications do these variations have for diets and the pigs consuming them? In vitro experiments demonstrated that rapid release of glucose contributed to the synchronized release of glucose and nitrogen in diets. Conversely, slower dietary glucose release disrupted this synchronization. Pigs fed diets with asynchronous release of dietary glucose and nitrogen exhibited lower apparent energy digestibility, reduced average daily gain, and increased fecal nitrogen excretion compared to those fed Syn diets. Our findings confirm previous studies that have observed the differential effects of synchronous versus asynchronous carbon and nitrogen supply in diets [1]. Furthermore, two types of diets with asynchronous release of glucose and nitrogen were identified in this study. We suggested that each type may result in lower daily gain of pigs through different mechanisms.

The first type of diet (Asyn_C: MGR_MGR) was characterized by an insufficient nitrogen supply throughout the digestion process. Previous study showed that amylose’s linear structure forms molecular entanglements with proteins, preventing protein aggregation, but excessive phase separation may promote protein self-aggregation and refolding, reducing digestibility [47]. Maltose, an intermediate in starch digestion, modifies the configuration of trypsin and reduces its activity, may also further impacting protein digestion [48]. Therefore, the reduced digestibility of crude protein and amino acids in MGR_MGR may be due to the persistent interaction between starch and protein, caused by slow starch digestion and glucose release. Moreover, pigs fed this diet exhibited the lowest ACE of ileal chyme among treatments, accompanied by a higher relative abundance of *Streptococcus* in the pigs’ terminal ileum when compared to the Syn. The genus *Streptococcus* includes over 50 species, and *Streptococcus suis* is one of the most serious and common pathogens affecting pigs [49–51]. Collectively, the delayed release of nitrogen and enrichment of harmful bacteria in the terminal ileum may be the reasons for the lower daily gain caused by such diets.

Another type of diet (Asyn_N: MSGR_MLGR, SGR_LGR) was characterized by an insufficient glucose supply. Different amino acids utilization was found in pigs fed this type of diet compared to those fed Asyn_C diet. Asyn_N showed higher apparent ileal digestibility (AID) for glutamate, isoleucine, leucine, and phenylalanine compared to the Syn. This increase may be attributed to the further reduced rate of glucose release in the Asyn_N diet, leading to a decrease in intermediates such as maltose that hinder protein digestion. Consequently, proteins digested in the gastric phase could be further broken down into amino acids. Moreover, in vivo microorganisms in the Asyn_N group appeared to prioritize carbon metabolism using amino acids. The earlier study showed that in the absence of fermentable carbohydrates as an energy source, nitrogenous compounds would be utilized, leading to an increase in end-products like NH3 [52]. Here we found a higher proportion of microbial communities involved in carbon metabolism and nitrogen-cycling-related processes, but a lower proportion involved in starch and sucrose metabolism. Despite further trials are needed to confirm and clarify the underlying mechanism, these findings support the idea that, in the presence of a limited glucose supply, excess amino acids may be converted into energy, potentially reducing protein deposition [53–55]. Additionally, it was also observed that compared to Syn group, Asyn_N group showed a higher relative abundance of *Streptococcus Enterococcus*, and *Actinobacillus* enriched in the pigs’ terminal ileum. *Enterococcus faecalis* and *Enterococcus faecium* are widely prevalent opportunistic pathogens that inhabit the intestinal tracts of animals [56, 57]. As previous study observed, branched-chain amino acids were essential for the survival and virulence of *Actinobacillus pleuropneumoniae* in swine [58]. Therefore, these findings suggest that the Asyn_N diet may negatively impact pig growth by increasing harmful bacteria and shifting the utilization of amino acids.

As for the Syn diets, it has neither a nitrogen deficit nor a glucose deficit throughout the digestive process. The eigengene of Module 7, which includes *Prevotella* and *Clostridium_sensu_stricto_1*, was higher in the Syn group. *Prevotella copri* has been reported to increase fat accumulation of pigs [59]. While *Clostridium butyricum* was observed that can slow down the effects of host fat deposition [60]. And *Clostridium butyricum* was found as a butyrate-producer to help convert lactate to butyrate, balancing the increased lactate production resulting from *Streptococcus* and *Enterococcus* fermentation of resistant starch [61–64]. Therefore, we suggest that the enrichment of *Prevotella* or *Clostridium_sensu_stricto_1* is not specifically attributed to diets with a beneficial synchronized release of glucose and nitrogen. However, the potential converse functions of these two microbes and their similar relative abundance in Module 7 may reflect a balance in carbon and nitrogen supply, which requires further confirmation.

Several limitations of the present study need to be addressed. To eliminate interference from dietary glucose addition levels and better simulate common feed diets, the experimental dietary glucose and nitrogen sources were derived solely from starch and protein, without additional supplementation as recommended by the National Research Council in diets of pigs fitted with T-cannula [65]. This may result in lower protein and amino acid utilization. Additionally, there were fluctuations in nitrogen and amino acid release at adjacent time points during the in vitro experiment, likely due to the low short-term release amounts. Therefore, our analysis focused on comparing relative differences rather than absolute values. Moreover, various kinetic models are used to explore nutrient digestion and release, but few can be universally applied [66, 67]. Thus, we used a direct calculation method instead. However, further experiments are needed to verify and optimize this method and the synchronization calculation method mentioned in this study for wider application.

## Conclusion

Comprehensive analysis of glucose release kinetics of twenty-three feed ingredients demonstrated that glucose release rate and amounts were extremely variable among feed ingredients. This variability, in turn, led to differences in growth performance among pigs fed diets containing different ingredients but having equivalent nutritional levels. Especially, our study revealed that slower glucose release disrupted the synchrony of dietary carbon and nitrogen supply, altering amino acid utilization, enriching pathogens, and ultimately slowed the growth of pigs and increased nitrogen losses. Conversely, the moderately rapid glucose release pattern achieved the synchronized and rapid release of glucose, soluble nitrogen, and amino acids. Pigs fed this diet had enhanced short-term growth performance, improved energy and nitrogen utilization, higher amino acid digestibility, and a decreased abundance of harmful bacteria such as *Streptococcus*. Thus, selecting feed ingredients releasing glucose at a rapid rate to balance dietary carbon and nitrogen supply holds great promise for promoting pig growth, ensuring efficient utilization of feed resources, and supporting sustainable agriculture.

## Supplementary Information

Additional file 1: Fig. S1. The correlation between glucose and nitrogen release rate. A The correlation between glucose and soluble nitrogen release rate at 0–20 min and 0–360 min during simulated intestinal phase. B The correlation between glucose and total amino acids release rate at 0–20 min and 0–360 min during simulated intestinal phase. R, Pearson correlation coefficient. *n* = 3 for each diet. Fig. S2. Response of ileal microbiome composition to asynchronous release of dietary glucose and nitrogen. A Boxplots of alpha diversity as measured by Abundance-based coverage estimator (ACE) of the ileal microbiome. B PCoA of the ileal microbiome based on the weighted Bray-Curtis distances metric. RGR_HGR, a diet that releases glucose rapidly and in large amounts; MRGR_MHGR, a diet that releases glucose at the second fastest rate and in the second largest amounts; MGR_MGR, a diet that releases glucose at a moderate rate and with moderate amounts of glucose release; MSGR_MLGR, a diet that releases glucose at the second slowest rate and with the second lowest amounts of glucose release; SGR_LGR, a diet that releases glucose slowly and with low amounts of glucose release; Syn, diets with better synchronous release of dietary glucose and nitrogen (RGR_HGR, MRGR_MHGR); Asyn, diets with asynchronous release of dietary glucose and nitrogen (MGR_MGR, MSGR_MLGR, SGR_LGR); No marking indicates there is no significance. *n* = 6 for each diet except RGR_HGR, where *n* = 5. Fig. S3. The difference in the relative abundance of members in Module 7 among treatments. RGR_HGR, a diet that releases glucose rapidly and in large amounts; MRGR_MHGR, a diet that releases glucose at the second fastest rate and in the second largest amounts; MGR_MGR, a diet that releases glucose at a moderate rate and with moderate amounts of glucose release; MSGR_MLGR, a diet that releases glucose at the second slowest rate and with the second lowest amounts of glucose release; SGR_LGR, a diet that releases glucose slowly and with low amounts of glucose release; Syn, diets with better synchronous release of dietary glucose and nitrogen (RGR_HGR, MRGR_MHGR); Asyn, diets with asynchronous release of dietary glucose and nitrogen (Asyn_C: MGR_MGR; Asyn_N: MSGR_MLGR, SGR_LGR); *n* = 6 for each diet except RGR_HGR, where *n* = 5. No marking indicates there is no significance. Fig. S4. The proportion of microbial communities involved in N cycling processes varied significantly among treatments. * indicates *P* < 0.05, ** indicates *P* < 0.01. *n* = 6 for each diet except RGR_HGR, where *n* = 5. RGR_HGR, a diet that releases glucose rapidly and in large amounts; MRGR_MHGR, a diet that releases glucose at the second fastest rate and in the second largest amounts; MGR_MGR, a diet that releases glucose at a moderate rate and with moderate amounts of glucose release; MSGR_MLGR, a diet that releases glucose at the second slowest rate and with the second lowest amounts of glucose release; SGR_LGR, a diet that releases glucose slowly and with low amounts of glucose release.

Additional file 2: Table S1. The chemical composition, place of origin of 23 feed ingredients samples (%, as-fed basis). Table S2. Analyzed chemical composition of the experimental diets (%, on a DM basis). Table S3. Effect of treatments on the apparent ileal digestibility (%) of amino acids. Table S4. Effect of treatments on the standardized ileal digestibility (%) of amino acids.

Additional file 3: Table S5. Analysis of metabolic functions of ASVs enriched in different treatments based on FAPROTAX.

## Data Availability

The datasets used and/or analyzed during the current study are available from the corresponding author on reasonable request.

## Abbreviations

AA: Amino acids
ACE: Abundance-based coverage estimator
ADG: Average daily gain
AID: Apparent ileal digestibility
ANOVA: Analysis of variance
AOAC: Association of Official Analytical Chemists
ASV: Amplicon sequence variants
ATTD: Apparent total tract digestibility
BW: Body weight
CP: Crude protein
DM: Dry matter
FDR: False discovery rate
FAPROTAX: Functional Annotation of Prokaryotic Taxa
GE: Gross energy
IAA: Basal ileal endogenous loss of an amino acid
PCoA: Principal coordinates analysis
PICRUSt2: Phylogenetic Investigation of Communities by Reconstruction of Unobserved States
PERMANOVA: Permutational multivariate analysis of variance
SID: Standardized ileal digestibility
SE-HPLC: Size exclusion-high performance liquid chromatography
