# Supplementary material for: Glucose release kinetics of different feed ingredients and their impact on short-term growth of pigs by influencing carbon-nitrogen supply synchronization
Source: J Anim Sci Biotechnol. 2025 May 22;16:72. doi: 10.1186/s40104-025-01198-6 (PMC12096610; doi:10.1186/s40104-025-01198-6)
Supplement: Supplementary file 1 — Additional file 1: Fig. S1. The correlation between glucose and nitrogen release rate. A The correlation between glucose and soluble nitrogen release rate at 0–20 min and 0–360 min during simulated intestinal phase. B The correlation between glucose and total amino acids release rate at 0–20 min and 0–360 min during simulated intestinal phase. R, Pearson correlation coefficient. n = 3 for each diet. Fig. S2. Response of ileal microbiome composition to asynchronous release of dietary glucose and nitrogen. A Boxplots of alpha diversity as measured by Abundance-based coverage estimator (ACE) of the ileal microbiome. B PCoA of the ileal microbiome based on the weighted Bray-Curtis distances metric. RGR_HGR, a diet that releases glucose rapidly and in large amounts; MRGR_MHGR, a diet that releases glucose at the second fastest rate and in the second largest amounts; MGR_MGR, a diet that releases glucose at a moderate rate and with moderate amounts of glucose release; MSGR_MLGR, a diet that releases glucose at the second slowest rate and with the second lowest amounts of glucose release; SGR_LGR, a diet that releases glucose slowly and with low amounts of glucose release; Syn, diets with better synchronous release of dietary glucose and nitrogen (RGR_HGR, MRGR_MHGR); Asyn, diets with asynchronous release of dietary glucose and nitrogen (MGR_MGR, MSGR_MLGR, SGR_LGR); No marking indicates there is no significance. n = 6 for each diet except RGR_HGR, where n = 5. Fig. S3. The difference in the relative abundance of members in Module 7 among treatments. RGR_HGR, a diet that releases glucose rapidly and in large amounts; MRGR_MHGR, a diet that releases glucose at the second fastest rate and in the second largest amounts; MGR_MGR, a diet that releases glucose at a moderate rate and with moderate amounts of glucose release; MSGR_MLGR, a diet that releases glucose at the second slowest rate and with the second lowest amounts of glucose release; SGR_LGR, a diet that releases gluco [file 40104_2025_1198_MOESM1_ESM.docx]

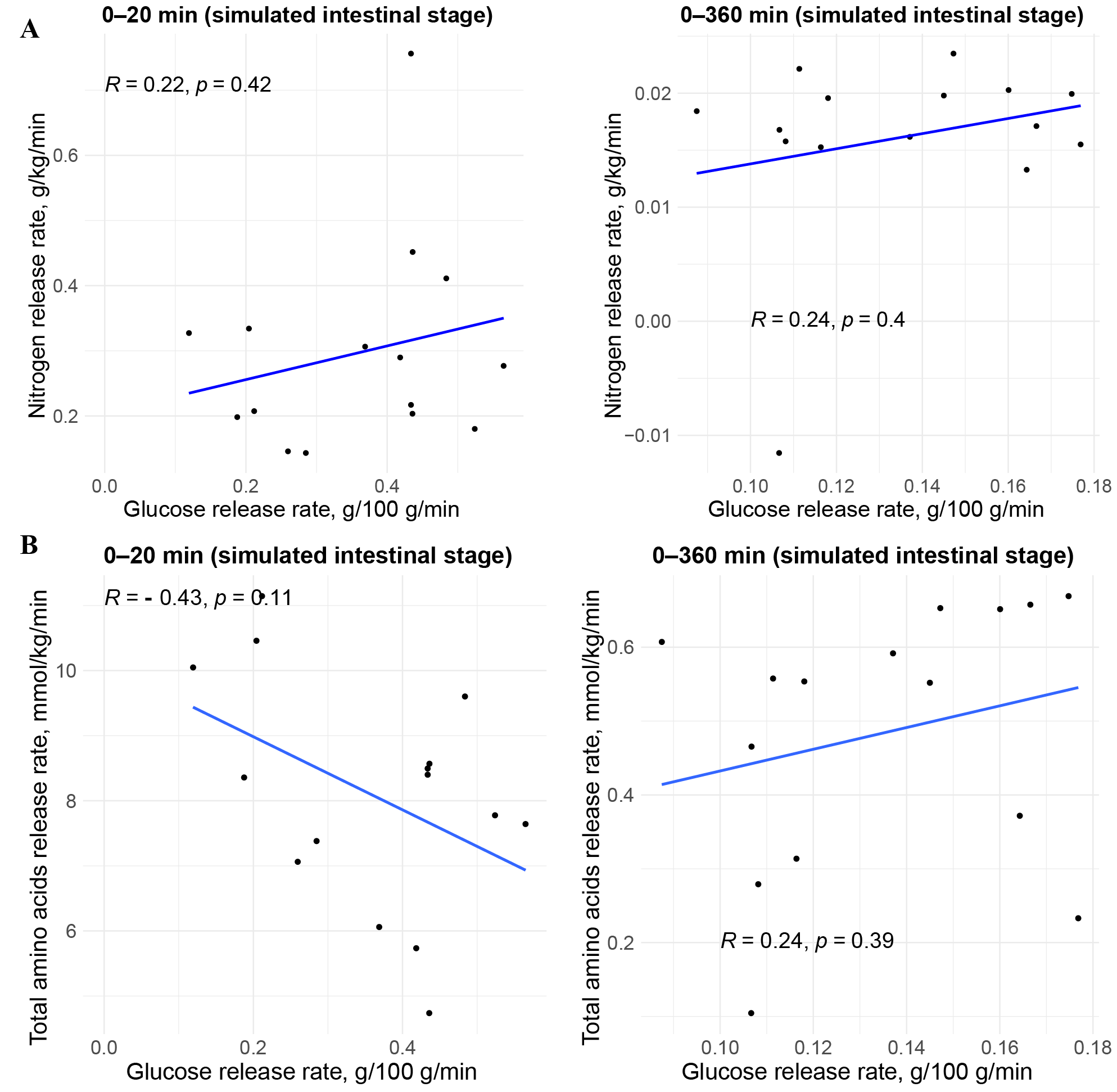


**Fig. S1** The correlation between glucose and nitrogen release rate. **A** The correlation between glucose and soluble nitrogen release rate at 0-20 min and 0-360 min during simulated intestinal phase. **B** The correlation between glucose and total amino acids release rate at 0-20 min and 0-360 min during simulated intestinal phase. R, Pearson correlation coefficient. *n* = 3 for each diet


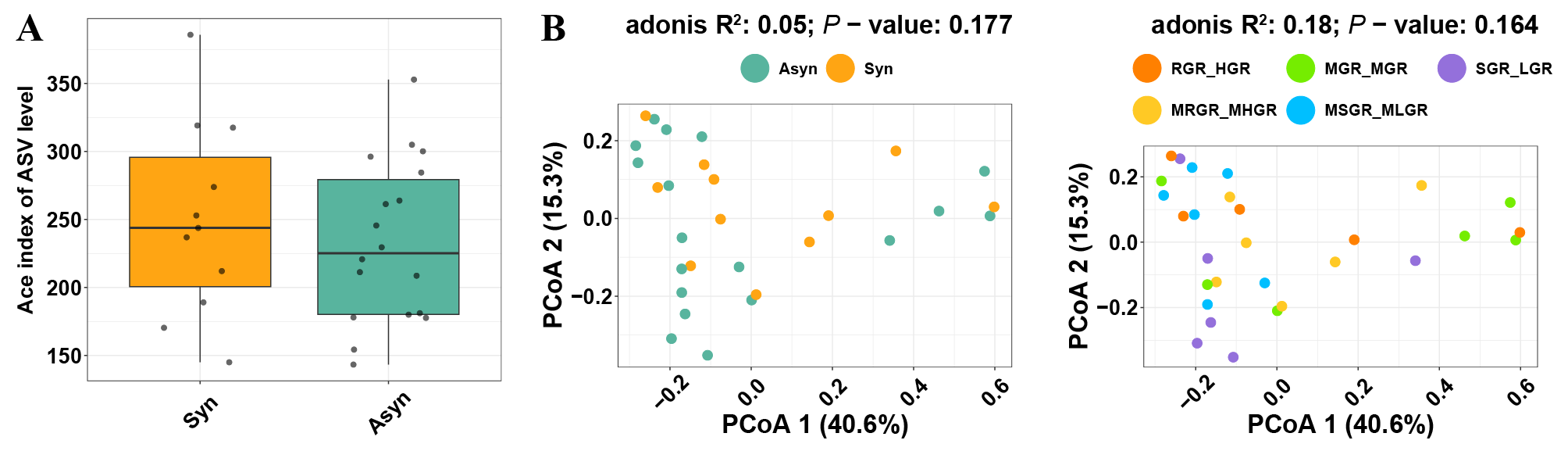


**Fig. S2** Response of ileal microbiome composition to asynchronous release of dietary glucose and nitrogen. **A** Boxplots of alpha diversity as measured by Abundance-based coverage estimator (ACE) of the ileal microbiome. **B** PCoA of the ileal microbiome based on the weighted Bray-Curtis distances metric. RGR_HGR, a diet that releases glucose rapidly and in large amounts; MRGR_MHGR, a diet that releases glucose at the second fastest rate and in the second largest amounts; MGR_MGR, a diet that releases glucose at a moderate rate and with moderate amounts of glucose release; MSGR_MLGR, a diet that releases glucose at the second slowest rate and with the second lowest amounts of glucose release; SGR_LGR, a diet that releases glucose slowly and with low amounts of glucose release; Syn, diets with better synchronous release of dietary glucose and nitrogen (RGR_HGR, MRGR_MHGR); Asyn, diets with asynchronous release of dietary glucose and nitrogen (MGR_MGR, MSGR_MLGR, SGR_LGR); No marking indicates there is no significance. *n* = 6 for each diet except RGR_HGR, where *n* = 5


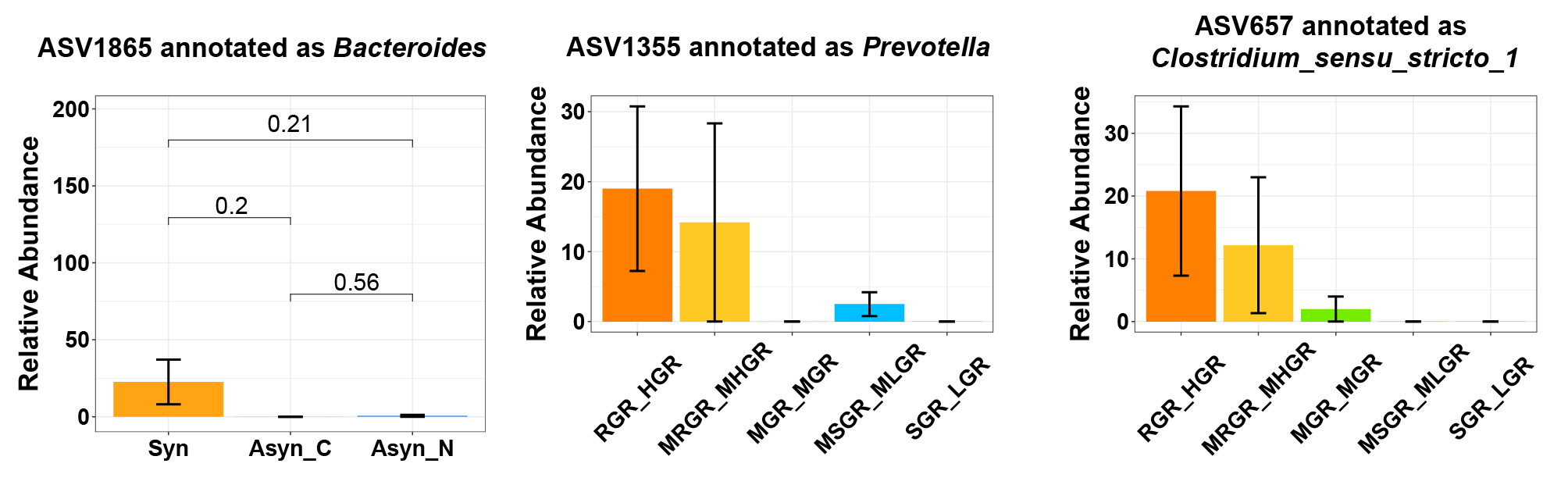


**Fig. S3** The difference in the relative abundance of members in Module 7 among treatments. RGR_HGR, a diet that releases glucose rapidly and in large amounts; MRGR_MHGR, a diet that releases glucose at the second fastest rate and in the second largest amounts; MGR_MGR, a diet that releases glucose at a moderate rate and with moderate amounts of glucose release; MSGR_MLGR, a diet that releases glucose at the second slowest rate and with the second lowest amounts of glucose release; SGR_LGR, a diet that releases glucose slowly and with low amounts of glucose release; Syn, diets with better synchronous release of dietary glucose and nitrogen (RGR_HGR, MRGR_MHGR); Asyn, diets with asynchronous release of dietary glucose and nitrogen (Asyn_C: MGR_MGR; Asyn_N: MSGR_MLGR, SGR_LGR); *n* = 6 for each diet except RGR_HGR, where *n* = 5. No marking indicates there is no significance


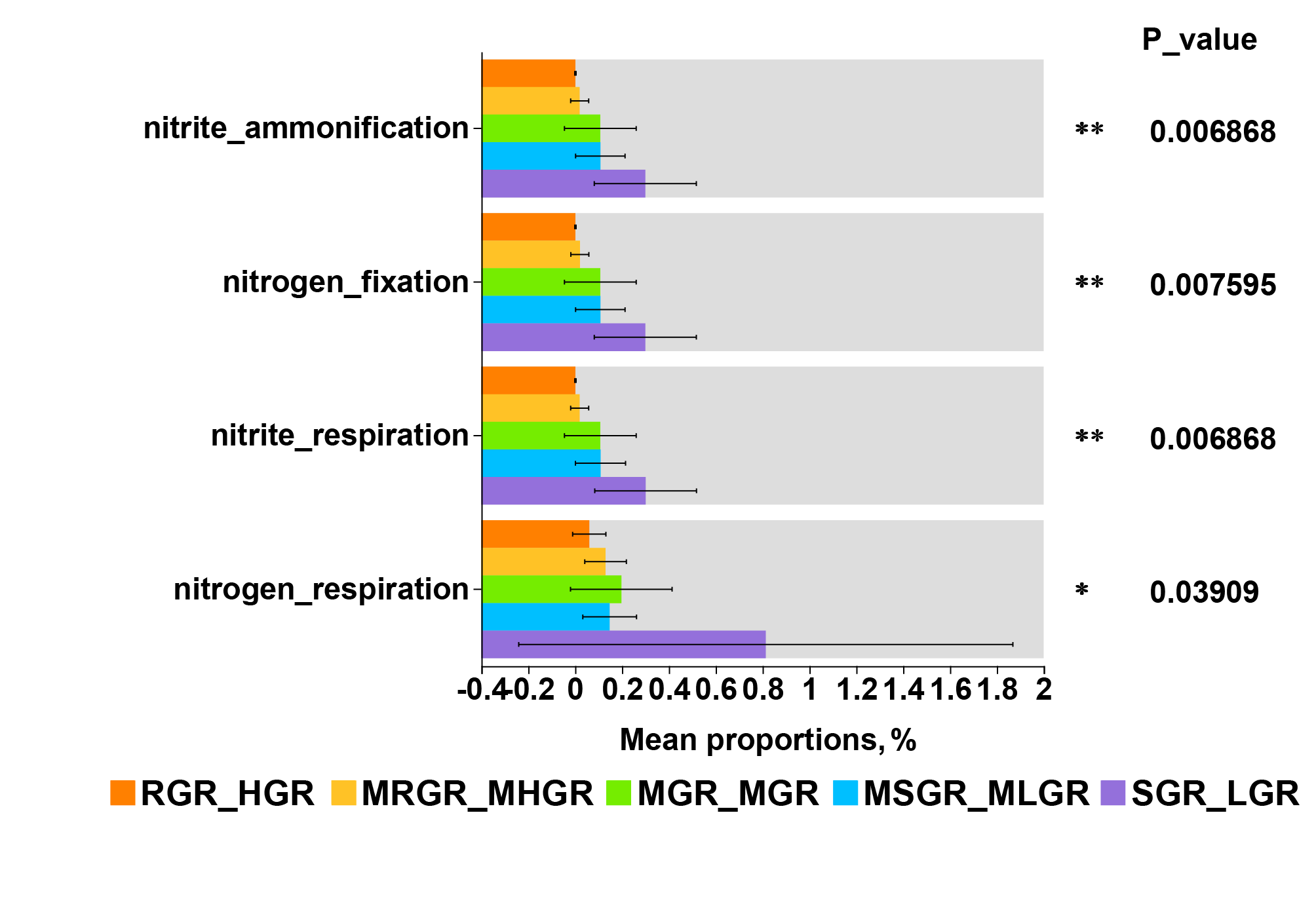


**Fig. S4** The proportion of microbial communities involved in N cycling processes varied significantly among treatments. * indicates *P* < 0.05, ** indicates *P* < 0.01. *n* = 6 for each diet except RGR_HGR, where *n* = 5. RGR_HGR, a diet that releases glucose rapidly and in large amounts; MRGR_MHGR, a diet that releases glucose at the second fastest rate and in the second largest amounts; MGR_MGR, a diet that releases glucose at a moderate rate and with moderate amounts of glucose release; MSGR_MLGR, a diet that releases glucose at the second slowest rate and with the second lowest amounts of glucose release; SGR_LGR, a diet that releases glucose slowly and with low amounts of glucose release
