# Supplementary material for: Glucose release kinetics of different feed ingredients and their impact on short-term growth of pigs by influencing carbon-nitrogen supply synchronization
Source: J Anim Sci Biotechnol. 2025 May 22;16:72. doi: 10.1186/s40104-025-01198-6 (PMC12096610; doi:10.1186/s40104-025-01198-6)
Supplement: Supplementary file 2 — Additional file 2: Table S1. The chemical composition, place of origin of 23 feed ingredients samples (%, as-fed basis). Table S2. Analyzed chemical composition of the experimental diets (%, on a DM basis). Table S3. Effect of treatments on the apparent ileal digestibility (%) of amino acids. Table S4. Effect of treatments on the standardized ileal digestibility (%) of amino acids. [file 40104_2025_1198_MOESM2_ESM.docx]

**Table S1** The chemical composition, place of origin of 23 feed ingredients samples (%, as-fed basis)

| **Ingredients** | **Place of origin** | **Starch, %** | **DM^1^, %** |
| --- | --- | --- | --- |
| Broken rice | Hunan, China | 79.01 | 87.82 |
| Brown rice | Jilin, China | 76.22 | 87.41 |
| Corn | Hebei, China | 68.80 | 87.85 |
| Wheat | Shandong, China | 62.71 | 88.82 |
| Sorghum | Australia | 61.01 | 87.99 |
| Barley | Belarus | 60.74 | 90.07 |
| Wheat middlings and red dog | Shandong, China | 41.18 | 89.45 |
| Rice bran | Beijing, China | 25.14 | 91.45 |
| Corn germ meal | Jilin, China | 22.17 | 91.40 |
| Defatted rice bran | Jilin, China | 19.64 | 90.73 |
| Wheat bran | Shandong, China | 13.25 | 90.36 |
| Palm Kernel meal | Heilongjiang, China | 2.32 | 92.18 |
| Corn gluten meal | Hunan, China | 16.72 | 92.30 |
| Peanut meal | Jilin, China | 14.82 | 90.67 |
| Rice protein | Guizhou, China | 7.94 | 91.91 |
| Corn DDGS | Heilongjiang, China | 5.67 | 91.47 |
| Soybean meal | Hebei, China | 4.92 | 89.31 |
| Cottonseed protein | Jilin, China | 3.62 | 93.41 |
| Fish meal | Guizhou, China | 1.96 | 92.08 |
| Milk casein | Gansu, China | 1.68 | 91.40 |
| Cottonseed meal | Xinjiang, China | 1.63 | 91.11 |
| Whey protein concentrate | USA | 1.54 | 91.43 |
| Sunflower meal | Ukraine | - | 91.62 |

^1^ *DM* Dry matter

**Table S2** Analyzed chemical composition of the experimental diets (%, on a DM basis)^1^

| **Items** | **RGR_HGR** | **MRGR_MHGR** | **MGR_MGR** | **MSGR_MLGR** | **SGR_LGR** | **NF** |
| --- | --- | --- | --- | --- | --- | --- |
| Arginine | 0.43 | 0.42 | 0.44 | 0.44 | 0.43 | 0.00 |
| Histidine | 0.15 | 0.14 | 0.15 | 0.14 | 0.14 | 0.00 |
| Isoleucine | 0.35 | 0.33 | 0.33 | 0.33 | 0.33 | 0.01 |
| Leucine | 0.53 | 0.53 | 0.53 | 0.53 | 0.53 | 0.04 |
| Lysine | 0.43 | 0.42 | 0.42 | 0.43 | 0.42 | 0.02 |
| Methionine | 0.12 | 0.13 | 0.14 | 0.12 | 0.13 | 0.06 |
| Phenylalanine | 0.26 | 0.26 | 0.27 | 0.26 | 0.26 | 0.02 |
| Threonine | 0.26 | 0.28 | 0.27 | 0.27 | 0.28 | 0.02 |
| Tryptophan | 0.11 | 0.12 | 0.11 | 0.12 | 0.12 | 0.21 |
| Valine | 0.37 | 0.37 | 0.35 | 0.36 | 0.36 | 0.03 |
| Alanine | 0.34 | 0.35 | 0.35 | 0.36 | 0.35 | 0.03 |
| Aspartic acid | 0.87 | 0.83 | 0.84 | 0.85 | 0.84 | 0.05 |
| Cystine | 0.13 | 0.15 | 0.14 | 0.15 | 0.14 | 0.00 |
| Glutamic acid | 1.15 | 1.20 | 1.21 | 1.20 | 1.20 | 0.19 |
| Glycine | 0.22 | 0.23 | 0.24 | 0.24 | 0.25 | 0.02 |
| Proline | 0.41 | 0.41 | 0.37 | 0.36 | 0.37 | - |
| Serine | 0.31 | 0.31 | 0.33 | 0.32 | 0.33 | 0.02 |
| Tyrosine | 0.17 | 0.18 | 0.17 | 0.18 | 0.17 | - |

^1^ All data are the results of chemical analysis conducted in duplicate

**Table S3** Effect of treatments on the apparent ileal digestibility (%) of amino acids^1^

| **Items** | **RGR_HGR** | **MRGR_MHGR** | **MGR_MGR** | **MSGR_MLGR** | **SGR_LGR** | **SEM** | ***P*** |
| --- | --- | --- | --- | --- | --- | --- | --- |
| Histidine | 83.65 | 76.88 | 74.66 | 74.80 | 82.95 | 1.38 | 0.09 |
| Lysine | 74.99 | 70.84 | 69.40 | 73.29 | 79.83 | 1.61 | 0.32 |
| Methionine | 86.53 | 82.01 | 83.85 | 82.40 | 85.02 | 0.83 | 0.60 |
| Threonine | 58.12 | 54.12 | 51.03 | 55.35 | 65.22 | 2.51 | 0.63 |
| Valine | 75.49 | 69.74 | 66.67 | 68.98 | 76.66 | 1.66 | 0.37 |
| Aspartic acid | 78.89 | 73.29 | 71.36 | 73.95 | 79.97 | 1.44 | 0.41 |
| Cystine | 75.03 | 67.15 | 63.26 | 64.66 | 72.53 | 1.82 | 0.28 |
| Glycine | -18.12 | -18.99 | -27.82 | -31.09 | 23.20 | 9.38 | 0.21 |
| Proline | -275.97 | -86.30 | -337.94 | -342.18 | -102.37 | 36.83 | 0.05 |
| Serine | 65.58 | 58.99 | 58.27 | 60.17 | 71.86 | 2.23 | 0.22 |

^1^ *P*, the *P* value of Kruskal-Wallis test among the RGR_HGR, MRGR_MHGR, MGR_MGR, MSGR_MLGR, SGR_LGR diets. *n* = 6 for each diet

**Table S4** Effect of treatments on the standardized ileal digestibility (%) of amino acids^1^

| **Items** | **RGR_HGR** | **MRGR_MHGR** | **MGR_MGR** | **MSGR_MLGR** | **SGR_LGR** | **SEM** | ***P*** |
| --- | --- | --- | --- | --- | --- | --- | --- |
| Arginine | 88.00 | 82.49 | 72.33 | 78.02 | 90.15 | 2.49 | 0.15 |
| Histidine | 89.70 | 83.23 | 80.76 | 81.11 | 89.28 | 1.38 | 0.1 |
| Isoleucine | 88.00 | 84.27 | 85.50 | 84.74 | 91.98 | 1.2 | 0.22 |
| Leucine | 87.01 | 83.10 | 81.27 | 82.88 | 91.26 | 1.46 | 0.21 |
| Lysine | 84.05 | 80.05 | 78.71 | 82.43 | 89.13 | 1.61 | 0.33 |
| Methionine | 92.18 | 87.41 | 88.77 | 88.14 | 90.30 | 0.83 | 0.56 |
| Phenylalanine | 87.24 | 81.36 | 80.83 | 80.90 | 92.85 | 1.73 | 0.08 |
| Threonine | 74.10 | 68.51 | 65.99 | 70.49 | 79.90 | 2.51 | 0.56 |
| Tryptophan | 72.62 | 71.08 | 63.60 | 75.97 | 75.69 | 2.03 | 0.36 |
| Valine | 86.41 | 80.78 | 78.16 | 80.05 | 87.83 | 1.65 | 0.39 |
| Alanine | 69.06 | 65.39 | 55.41 | 61.59 | 77.76 | 3.61 | 0.32 |
| Aspartic acid | 86.62 | 81.36 | 79.33 | 81.86 | 87.94 | 1.44 | 0.4 |
| Cystine | 84.82 | 75.88 | 72.35 | 73.53 | 81.55 | 1.84 | 0.23 |
| Glutamic acid | 90.93 | 91.96 | 86.32 | 87.95 | 95.20 | 1.2 | 0.19 |
| Glycine | 20.17 | 17.85 | 7.93 | 3.93 | 57.41 | 9.34 | 0.21 |
| Proline | (201.07) | (11.25) | (253.49) | (256.28) | (18.89) | 36.61 | 0.07 |
| Serine | 77.88 | 71.23 | 69.54 | 71.86 | 83.24 | 2.23 | 0.28 |
| Tyrosine | 85.49 | 86.29 | 78.15 | 88.49 | 96.18 | 2.1 | 0.09 |

^1^ *P*, the *P* value of Kruskal-Wallis test among the RGR_HGR, MRGR_MHGR, MGR_MGR, MSGR_MLGR, SGR_LGR diets. *n* = 6 for each diet
